# Primary Study Quality in Psychological Meta-Analyses: An Empirical Assessment of Recent Practice

**DOI:** 10.3389/fpsyg.2018.02667

**Published:** 2019-01-09

**Authors:** Richard E. Hohn, Kathleen L. Slaney, Donna Tafreshi

**Affiliations:** Department of Psychology, Simon Fraser University, Burnaby, BC, Canada

**Keywords:** meta-analysis, meta-analysis reporting standards (MARS), reporting practices, study quality, risk of bias, primary study quality, study quality assessment, study quality assessment criteria

## Abstract

As meta-analytic research has come to occupy a sizeable contingent of published work in the psychological sciences, clarity in the reporting of such work is crucial to its interpretability and reproducibility. This is especially true regarding the assessment of primary study quality, as notions of study quality can vary across research domains. The present study examines the general state of reporting practices related to primary study quality in a sample of 382 published psychological meta-analyses, as well as the reporting decisions and motivations of the authors that published them. Our findings suggest adherence to reporting standards has remained poor for assessments of primary study quality and that the discipline remains inconsistent in its reporting practices generally. We discuss several potential reasons for the poor adherence to reporting standards in our sample, including whether quality assessments are being conducted in the first place, whether standards are well-known within the discipline, and the potential conflation of assessing primary study quality with other facets of conducting a meta-analysis. The implications of suboptimal reporting practices related to primary study quality are discussed.

## Introduction

Meta-analysis has come to occupy a significant role in many research disciplines, especially within the medical and social sciences. Given the advantages that meta-analytic techniques present relative to other methods of research synthesis, the rise in their popularity comes as no surprise. For example, meta-analytic techniques enable the synthesis of larger and more representative samples compared to isolated primary studies, allow researchers to explore the influence of moderator variables on the between-study variability of primary study results (i.e., effect sizes), and facilitate the estimation of the degree to which the primary study results are homogeneous. Importantly, meta-analysis is thought to offer a systematic approach toward the mitigation of multiple sources of subjective biases considered to be present in traditional narrative literature reviews.

As a consequence of the accelerated growth in popularity of meta-analysis, a number of empirical studies have examined the reporting and methodological practices in published psychological meta-analyses (Harwell and Maeda, 2008; Dieckmann et al., 2009; Aytug et al., 2012; Brugha et al., 2012; Slaney et al., 2018). Common among these reports are findings that suggest reporting practices are often insufficient and decentralized, both across and within research areas. For example, within organizational psychology, Aytug et al. (2012) determined that meta-analyses published between 1995 and 2008 scarcely reported the amount of information sufficient for replication. Similar findings were reported by Harwell and Maeda (2008) and Dieckmann et al. (2009) regarding meta-analyses in general psychological and educational research, respectively. Moreover, Brugha et al. (2012) concluded that reporting practices for psychiatric meta-analyses of observational studies published between 1996 and 2009 were deficient. Unanimously, these examinations of reporting practices called for the adoption of either localized or discipline-wide reporting standards to promote more transparent, consistent, and replicable meta-analyses.

Resources and standards for formulating, conducting, and reporting meta-analyses have since begun to appear in both organization-specific and interdisciplinary contexts. Perhaps most popular has been the publication of several accessible introductory works, such as Borenstein et al. (2009), Cooper et al. (2009), and Cooper (2017). Numerous organizational bodies have specified their own standards for meta-analysis, primarily in reference to the reporting practices of meta-analytical research. Most notable among these is the *Cochrane Handbook for Systematic Reviews of Interventions* (CH; Higgins and Green, 2011), which thoroughly describes methodological and reporting criteria necessary for systematic reviews to be included in the Cochrane Collaboration’s curated database. Other established reporting standards are those included in the Methodological Expectations of Cochrane Intervention Reviews (MECIR; Higgins et al., 2017), the Quality of Reporting of Meta-Analyses (QUOROM) statement (Moher et al., 1999), the Preferred Reporting Items for Systematic Reviews and Meta-Analyses (PRISMA; Moher et al., 2009), the Meta-Analysis of Observational Studies in Epidemiology reporting proposal (MOOSE; Stroup et al., 2000), Transparent Reporting of Evaluations with Non-randomized Designs (TRENDs) Statement (Des Jarlais et al., 2004), and the Meta-Analysis Reporting Standards (MARS; American Psychological Association [APA] Publications and Communications Board Working Group on Journal Article Reporting Standards, 2008). With the exception of the CH, these standards come in the form of prescriptive lists that provide minimum criteria for what should be described within the various sections of meta-analytic reports. The decision of which reporting standards to reference can depend on multiple factors, such as what discipline the meta-analysis applies to (e.g., psychology, epidemiology) or the features of the individual studies synthesized in the meta-analysis (e.g., intervention studies vs. non-intervention studies).

The aim of adopting reporting standards is to ensure that any and all information relevant to meta-analytic studies be included in published works, such that consumers of the results of meta-analytic research may judge the validity of conclusions drawn in an accurate and well-informed manner. Of critical importance is the reporting of the many methodological decisions that accompany the implementation of statistical procedures and presentation of results of a meta-analysis. These include the methods are employed to search for primary studies, procedures and justifications for excluding primary studies from analysis, and the assessment of the quality of primary studies. Although the specification of reporting standards is a clear step toward the production of interpretable and replicable meta-analyses, a number of problems continue to impede such progress. The most apparent issue is that the usefulness of reporting standards depends on whether they are properly implemented in practice. Moreover, reporting standards tend to be vague regarding the level of detail required (e.g., “report study quality assessment”). Yet another problem is that, at present, it is not clear that adherence to any particular set of reporting standards is consistent within research disciplines and subdisciplines (e.g., clinical psychology, educational psychology, social psychology, etc.).

Several studies have demonstrated that adherence to reporting standards is lacking in both medical and behavioral research domains with respect to a variety of facets of conducting a meta-analysis. For example, Mullins et al. (2014) found that, for meta-analyses related to HIV behavioral interventions, details regarding the search methods used to obtain a sample of primary study articles were often underreported, despite search methods appearing in all reporting standards. Likewise, Tsou and Treadwell (2016) found that, for systematic reviews and meta-analyses of interventions, abstracts were oftentimes unclear, averaging an adherence rate of only 60% to the PRIMSA-A, a reporting standard document for abstracts. Poor adherence rates for the PRIMSA were also observed in large sample of otorhinolaryngologic reviews and meta-analyses by Peters et al. (2015). In a content analysis comparing the QUOROM statement to 24 other reporting standards, Shea et al. (2001) noted that none of the 24 standards were as comprehensive as the QUOROM, with most standards containing similar content for methods-based items, but variable overlap with the QUOROM in other areas of meta-analytic reporting (e.g., abstracts, results, discussions). Further, when a pilot sample of four meta-analyses were evaluated in light of six of the 24 standards, Shea and colleagues found each set of reporting standards produced a different rank order of the meta-analyses with respect to their reporting qualities. More recently, Panic et al. (2013) observed that journal endorsements of the PRISMA resulted in increases in reporting and methodological quality of meta-analyses for gastroenterological- and hepatological-related meta-analyses. ^1^ For psychological meta-analyses, Slaney et al. (2018) observed that while reporting rates for statistical model choice was found to be high, researchers most often did not report the rationales or justifications for their choice, despite the appearance of this reporting standard in the MARS. Atkinson et al. (2015) outlined the lack of detail provided by the PRISMA, MARS, MECIR, and other standards for reporting items related to primary study search methods.

As mentioned above, among the consequences of suboptimal reporting practices is the distortion of interpretability of meta-analytic studies as well as increased difficulty of replication. These issues are only exacerbated when reporting practices are poor for those facets of conducting a meta-analysis for which a consensus approach has not been reached. For these, variability in conceptual understandings can lead to variability in the strategies employed to conduct or assess them. The present study focuses on one such facet of conducting a meta-analysis, namely, the practices associated with reporting and conducting assessments of *primary* *study quality* (PSQ).^2^

Debates related to PSQ have persisted in meta-analytical work for as long as the term “meta-analysis” itself has existed (see Glass, 1976, 2000; Smith and Glass, 1977; Eysenck, 1978; Chow, 1986; Sharpe, 1997) and appeals have long been made to synthesis researchers that they give more attention to concerns of PSQ (see Hedges, 1990). Reaching a consensus definition for the term “study quality” is not a straightforward endeavor, as notions of PSQ are often dependent upon the aims of the primary study in question within a particular research domain (Valentine, 2009). Still, a number of general definitions have been offered, such as Valentine’s (2009) description of PSQ as “the fit between a study’s goals and the study’s design and the implementation characteristics” (p. 131), and Cooper’s (2017) definition of PSQ as reflecting “the degree to which the study’s design and implementation permit you to draw inferences that guide your work” (p. 256). Although both definitions are quite general, they each reference PSQ as an issue related to study design and implementation. In addition, both definitions treat PSQ as an indicator of the validity of inferences drawn from a primary study and, by extension, any research synthesis of a set of primary studies.

A more standardized notion of PSQ has been offered in the CH that focuses on assessing potential sources of bias that may systematically impact the verisimilitude of a meta-analytic finding. The *risk of bias* approach is differentiated conceptually from PSQ in the CH primarily on the grounds that the PSQ concept remains ambiguous and that even primary studies which might otherwise be considered of high methodological PSQ can still contain systematic biases. To reference an example given by Higgins and Green (2011), imagine a scenario in which a researcher is testing the mean difference between a control group and a treatment group on some dependent measure. Suppose, for whatever reason, that blinding the participants of the study to which group they belong to is infeasible. All other considerations held constant, such a study could be judged to be of high PSQ, insofar as the best methodological procedures as possible were enacted. Yet, regardless of the inability of blinding participants, the failure to do so still represents a potential source of bias. Thus, in this scenario the judgment of the study relative to the blinding of participants is incongruent between the PSQ and risk of bias conceptualizations. In alignment with the Cochrane Collaboration’s focus on medical intervention research, the conceptualization and approach to assessing the risk of bias for studies included in a meta-analysis primarily concern randomized controlled trials (RCTs). At first glance, this seems to indicate that the risk of bias approach is not applicable to many psychological meta-analyses, given the vast employment of quasi-experimental and observational designs within many domains of psychological research. Yet, in the most recent update of the MARS, risk of bias language has replaced all references to PSQ (Appelbaum et al., 2018). For brevity, we include reference to risk of bias assessments when discussing PSQ assessments more broadly and make explicit conceptual distinctions where necessary.

At present, there are many proposed ways in which PSQ may be assessed. As early as 1995, at least 34 assessment tools (25 scales and 14 checklists) had been developed to assess the quality of RCTs (Moher et al., 1995) and newer PSQ assessment tools continue to develop, such as the more recently proposed Study Design and Implementation Assessment Device (Valentine and Cooper, 2008). Assessment tools have also been developed explicitly for evaluating the PSQ of quasi-experimental and observational studies, such as the Newcastle-Ottawa Scale (NOS; Wells et al., 2009), Quality Assessment of Diagnostic Accuracy Studies (QUADAS; Whiting et al., 2011), and the so-called Downs and Black Checklist (Downs and Black, 1998), to name only a few. Numerous systematic reviews of PSQ assessment tools have found 100s of unique tools used within the domain of medical research (Conn and Rantz, 2003; Deeks et al., 2003). As Conn and Rantz (2003) comment, however, the creation of PSQ assessment tools has seldom led to the employment of “established scale development techniques,” such as evaluations of reliability and validity (p. 324). More specifically, Conn and Rantz cite the Jadad scale (Jadad et al., 1996), QUOROM, Downs and Black Checklist, and a tool created by Sindhu et al. (1997) as the only four PSQ assessment tools to have been developed using established psychometric techniques at the time of their study’s publication in 2003. Deeks et al. (2003) found that only 14 of 193 examined tools for assessing the PSQ of non-randomized studies met the criteria necessary to be considered comprehensive and that most tools neglected features related to the internal validity of non-randomized studies.

Whether a published PSQ assessment tool is employed or some other coding scheme for assessing PSQ is utilized, the results of PSQ assessments are often used to inform strategies for incorporating PSQ considerations into the findings or interpretations of a meta-analysis. Such strategies include *a priori* methods, such as treating PSQ as a criterion for excluding primary research studies or using PSQ as a factor in determining a weighting scheme for the primary study results, as well as *post hoc* methods that treat PSQ as an empirical question regarding how the results of a meta-analysis might have been affected. One example of a popular *post hoc* method is when quality is treated as a moderator of effect sizes. In doing so, researchers are able to use a data-driven approach toward inferring the effect of PSQ on the magnitude and directionality of effect sizes in addition to deriving a quantitative estimate of how PSQ may have impacted the overall summary effect. Of course, none of these strategies are without disadvantages. The use of PSQ as a criterion for exclusion can lead to exclusion rules that may be interpreted as arbitrary, given that such rules are based on some predetermined threshold of PSQ as defined by the researcher or assessment tool. Unsurprisingly, establishing a predetermined threshold of PSQ invites a potentially high degree of researcher subjectivity, the mitigation of which requires a higher amount of empirical and theoretical support than is typically reported in published meta-analyses (Valentine, 2009). Likewise, the use of PSQ scores has been met with criticism by some who charge that single-value measures of PSQ distort the multifaceted dimensions of PSQ and ultimately lead to biased estimations of treatment effects when used as part of a weighting scheme (Greenland, 1994a,b; Greenland and O’Rourke, 2001; Herbison et al., 2006; Valentine and Cooper, 2008). Additionally, Jüni et al. (1999) found that the 25 published PSQ assessment scales described by Moher et al. (1995), when applied to the same sample of primary studies, produced disparate summary effects, leaving the validity of the conclusions drawn from the meta-analyses to be suspect (see also Cooper, 2017). Jüni et al. (1999) findings bear on analyses of PSQ moderators as well, as the outcomes of such analyses will be influenced by how PSQ is judged given the assessment tool used to obtain categories of PSQ (e.g., low-, medium-, high-quality) or continuous measures of PSQ (e.g., quality scores). Herbison et al. (2006) extended the work of Jüni et al. (1999), finding similar results when comparing quality scores from 45 PSQ assessment scales on a sample of meta-analyses extracted from the Cochrane Library.

Taken together, the above considerations suggest that understanding and assessing PSQ, as well as the actions taken as a result of PSQ assessments, comprise a potentially precarious facet of conducting meta-analyses. This may be especially true within psychological research, in which the assessment of PSQ is a complex task given the discipline’s proclivity for using quasi-experimental and observational study designs. It is thus an open question as to whether researchers in psychology are addressing PSQ adequately in their meta-analyses.

The broad aim of the present study is to explore the facet of PSQ in psychological meta-analyses. To facilitate this aim, a two-phase approach was employed in which data from two sources were collected and analyzed. First, a sample of 382 published psychological meta-analyses were reviewed and coded for items related to PSQ, such as whether an assessment of PSQ was reported, the stated purposes of assessing PSQ, and by what criteria PSQ was assessed. The objective of this phase was to capture the general state of reporting practices related to PSQ. Next, the first authors of the meta-analyses from the coding sample were contacted and surveyed directly. The survey queried authors as to whether they assessed PSQ, regardless of whether their assessments were ultimately reported in the published meta-analysis. It also contained items related to other quality-specific issues and gave authors the opportunity to comment on a broader set of concerns related to their meta-analysis research experiences, such as which resources (i.e., textbooks, reporting standards) they consulted when conducting and reporting their meta-analysis. As such, information related to *practices* of PSQ assessment was obtained, beyond what the article coding protocol from the first phase of the current project was capable of detecting. The two phases described above are referred to as “Coding” and “Survey” in the sections that follow.

## Materials and Methods

### Samples

#### Coding

**FIGURE 1:**
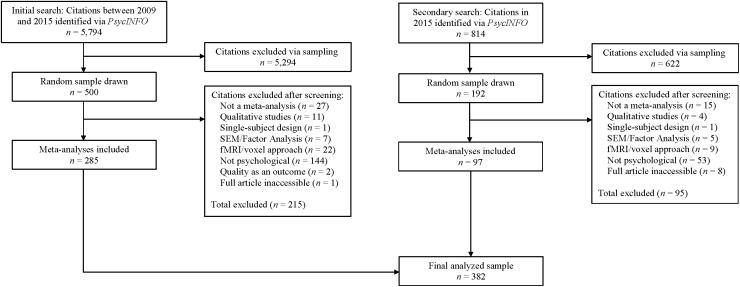
Selection and exclusion of published meta-analyses. Note that the frequencies for “Citations excluded after screening” represent rough estimates for each exclusion criterion. At the time the samples were compiled the frequencies of exclusion for each criterion were not recorded. At the suggestion of a reviewer of this article, we attempted to obtain the frequencies *post hoc* by coding each excluded article for the criterion we judged most likely to be the reason it was excluded. The coding procedure was completed only by the first author of the present study.

The *N* = 382 articles in our sample were obtained through searches of the *PsycINFO*© database. The searches were performed using the following criteria: (a) peer-reviewed journal articles, (b) “meta-analysis” used as an empirical method, and (c) publication date between 2009 and 2015. The initial search resulted in a total of 5,794 articles. From this initial sample, 500 articles were randomly selected using the random number generator in Microsoft Excel (i.e., the articles listed by the generator as the first 500 were selected for the sample). Each of the 500 articles was reviewed and exclusion criteria were applied. A secondary search was conducted to incorporate more articles into the sample and followed similar procedures as the initial search. Non-meta-analyses and articles that used qualitative analytic techniques were excluded. In addition, meta-analyses of voxel, fMRI, or other specifically neuroscientific studies were excluded, as well as studies that predominantly employed structural equation modeling or factor analytic techniques. Articles that involved single-subject designs or that were not directly related to the field of psychology were also excluded. Finally, articles that explored PSQ as an outcome variable were excluded from the final sample. After exclusions, the final sample consisted of *N =* 382 articles (see Figure 1). Thirty-two of these articles were published in 2009, 36 in 2010, 61 in 2011, 48 in 2012, 58 in 2013, 55 in 2014, and 97 in 2015.

#### Survey

For each article in the final sample, the contact information of the first author was collected and an invitation to the survey was distributed via e-mail. Participant invitations containing a description of the project, a citation of their meta-analysis, and link to the survey were sent to all first authors using the email invitation feature included in LimeSurvey’s software (LimeSurvey GmbH, 2017). In the event that a researcher was a first author for multiple papers within our sample of articles, a random number generator in Excel was used to select which of their meta-analyses would be indicated as the target meta-analysis of interest and authors were asked to respond only to the decisions made for the chosen meta-analysis. Sixty-nine invitations were undelivered due to issues with recipient email servers, email addresses that no longer existed, and other technical issues beyond the ability of our research team to troubleshoot. For any outdated email addresses, attempts were made to retrieve the authors’ updated email addresses and to resend invitations. Several first authors replied to the invitation and advised that we contact one of the other authors of their meta-analyses. In such cases, the recommendations of the first authors were followed and new invitations were sent to the suggested authors. The survey was available online for 8 weeks. Eighty-six authors responded to all or part of the survey.

### Materials

#### Coding

Each of the 382 articles in the sample was coded using the Quality Assessment for Systematic Reviews-Revised, or QUASR-R (Slaney et al., unpublished). The QUASR-R consists of six sections that address the various facets of conducting a meta-analysis, including which journal a meta-analysis article is published in, the search methods employed, exclusion/inclusion criteria specified^3^, methods related to interrater agreement and power used, methods related to model choice and characteristics coding used, and how PSQ was addressed. Of primary concern in the present study are the items in the PSQ section of the QUASR-R, which assess the following issues: (a) whether articles reported PSQ assessments; (b) whether they explicitly described their assessment method (e.g., coding protocol, quality scale used); (c) the stated purpose(s) for which PSQ was assessed; (d) the stated criteria used to judge PSQ; (e) whether the importance of assessing PSQ was explicitly addressed; (f) whether authors provided rationales or justifications for *not* reporting PSQ assessments. For (f), we extracted relevant excerpts from the articles in an attempt to gain insight as to why PSQ might not be assessed by those authors. Also relevant to the present study were items we hypothesized might practically or conceptually overlap with notions of PSQ and other demographic items. These additional items coded for whether study design characteristics were reported as well as the primary research aims of the meta-analyses (e.g., intervention, review, psychometric, replication). In response to reviewer suggestions the first author also coded for items related to heterogeneity analysis that did not appear in the QUASR-R.

#### Survey

The survey was constructed as part of a larger data collection effort related to investigating meta-analytic conduct and reporting practices in psychology. The primary aim of the survey was to allow authors to contextualize the methodological and reporting decisions they encountered when conducting their meta-analyses. As such, the survey elaborated and extended the subject matter coded by the QUASR-R. The survey consisted of primarily nominally scaled items (e.g., yes or no, checkbox endorsement). For some items, authors were asked to supplement their nominal responses with open-response comments or rationales relating to the item. As it regards the present investigation, items pertaining to authors’ assessments and conceptions of PSQ as well as their purposes for assessing PSQ were most relevant. Also relevant were items that invited researchers to indicate the resources (e.g., textbooks, reporting standards) they consulted while conducting their meta-analyses and to further discuss the availability and usefulness of those resources.

### Procedure

#### Coding

Articles were coded by research assistants and the third author of the current study. The research assistants were senior undergraduate students in psychology who were trained in reading and interpreting the methods and results sections of meta-analytic studies. To establish inter-rater reliability, 30% of the articles were initially coded by two research assistants. Coders independently familiarized themselves with articles and also independently assigned codes. Coders met with the third author on a bi-weekly basis to discuss their pre-assigned codes and to resolve discrepancies. Inter-rater agreement was greater than 70% for all items involved in the current analyses. Inter-rater agreements and kappa coefficients for all items are found in Table 1. Once inter-rater reliability was established for the full set of items, the remaining 70% of articles were divided into two halves, each of which was coded independently by a single undergraduate research assistant. As before, any coding issues or uncertainties were discussed and resolved with the third author.

**Table 1:** Inter-rater reliability for the QUASR-R items.

QUASR-R items	%	κ	95% CI
Reported assessment of PSQ	90.0	0.688	(0.522, 0.853)
Coding method described	86.7	0.704	(0.560, 0.849)
Purpose for assessing PSQ	77.8	0.601	(0.469, 0.870)
Discussed the importance of assessing PSQ^a^	82.2	0.545	(0.260, 0.831)
Reported criteria used to judge quality			
Study design	91.1	0.744	(0.501, 0.986)
Missing data or attrition	91.1	0.818	(0.644, 0.992)
Sample size	100	1.00	–
Measured used	82.2	0.643	(0.416, 0.870)
Statistical analyses conducted	84.4	0.632	(0.380, 0.884)
Literature review	100	1.00	–
Adequacy of study conclusions	86.7	0.182	(–0.244, 0.608)
Definition/operationalism of	97.8	0.789	(0.377, 1.00)
constructs			
Publication status	93.3	0.378	(–0.170, 0.925)
Power	100	1.00	–
Validity	93.3	0.690	(0.352, 1.00)
Reliability	97.8	0.656	(0.014, 1.00)
Reported study design characteristics	72.5	0.393	(0.219, 0.566)
Research aim	85.1	0.719	(0.599, 0.839)


*^a^Such as quality’s impact on the validity of conclusions drawn by the meta-analysis or primary studies, or the potential limitations resulting from the quality of primary studies.*

For the item that coded whether authors reported PSQ assessments, coders were instructed to search for the keyword “quality” within each article in order to aid in their determination of whether an assessment was reported. A consequence of this coding restriction is that articles in which the term “quality” was not used in explicit reference to PSQ could have been coded as not having reported an assessment. During the data analysis stage, the current study’s first author searched all articles in the sample for the phrase “risk of bias” to further determine if the CH’s risk of bias conceptualization was represented in a way that was not detected during the initial QUASR-R coding phase.^4^ Additionally, a number of QUASR-R items coded for which criteria were reportedly used to judge PSQ (e.g., study design, sample size, measures used, etc.). The criteria coded for by these items were chosen as a result of reviewing a subset of articles that reported assessing PSQ. In the event that new criteria were observed, they were added to the coding procedure.

#### Survey

Authors were invited to complete the online survey via personal e-mail communication.Authors were provided a description of the study objectives which explained the intentions of the survey as seeking their input as a collaborative effort to understand the decisions, obstacles, and influences that *actual researchers* encounter when conducting a meta-analysis. Authors were assured that the intention of the survey was not to test their knowledge of meta-analytic practices or assess the merits of their meta-analyses. Further, authors were made aware that their responses would remain confidential and that no information would be shared that could connect their survey responses to their meta-analyses. Before proceeding to the survey, participants were required to acknowledge their informed consent. Participants were informed that participation was voluntary and no incentives were offered for participation. Responses to individual items were also voluntary, such that authors were free to skip items and navigate the survey at their own discretion. Consequently, sample sizes vary among the items. No time limits were imposed for responding, although the survey was designed to be completed within 30 min. The survey was adaptive to participant responses. Where appropriate, participants were asked follow-up questions dependent on their responses to previous items. For example, if a participant author indicated that they assessed PSQ in their meta-analysis, they were then given follow-up questions related to PSQ. Upon completion of the survey, participants were thanked for their participation.

## Results

### Reporting and Assessing Primary Study Quality

#### Coding

Using the coding procedures described above, we found that of the 382 articles in our sample, only 117 (30.6%) explicitly reported that PSQ was assessed in some way. Of those 117, 64 (54.7%) articles explicitly described the methods used to assess PSQ. Thirty-three (28.2%) of the 117 articles discussed issues relating to PSQ, such as whether the outcomes of their assessments affected the validity of their conclusions, any resultant limitations, or the importance of assessing PSQ in the context of their meta-analysis. Moreover, of the 265 articles that did not explicitly report PSQ assessments, 16 (6%) provided a rationale or justification as to why PSQ was not assessed.^5^ In review of relevant excerpts extracted from those 16 articles, authors stated several different reasons for why PSQ was not assessed, including the desire to retain a large sample size in their meta-analysis, that the assessment of PSQ was unnecessary because stringent inclusion criteria were used, because publication bias and heterogeneity were addressed, or because detailed information about each of the studies included in the meta-analysis was available as supplementary material online and, therefore, accessible to readers of the reported meta-analysis. Due to the possibility that important information about PSQ assessment might have been expressed in terms of the risk of bias concept, the first author of the current study searched for instances of “risk of bias” in the full sample of articles and identified 35 articles containing the phrase. For these articles, we examined responses for the QUASR-R item that coded whether a PSQ assessment was reported. Twenty-eight of the 35 articles had been previously coded as having reported PSQ assessments. The remaining seven articles were recoded for this *QUASR-R* item. Three articles that reported risk of bias assessments were identified and subsequently recoded as having reported PSQ assessments. Additionally, those three articles were recoded for all quality-related QUASR-R items. The above findings and all subsequent findings incorporate the data from these three articles.

#### Survey

Authors were first asked whether they had assessed PSQ in their meta-analyses. Of the 71 authors that responded to this item, 43 (60.6%) indicated that they had assessed PSQ. Cross-referencing those 43 articles with their corresponding QUASR-R results revealed that 16 (37.2%) were coded as having reported a PSQ assessment, 12 of which provided at least some description of *how* PSQ was assessed and only three of which discussed issues relating to PSQ in their published articles.

### Purposes for Assessing Primary Study Quality

#### Coding

**FIGURE 2:**
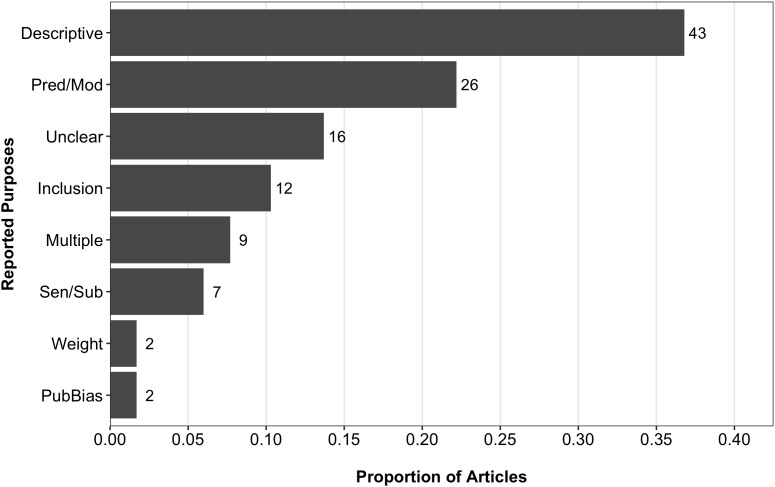
Proportions and frequencies of reported purposes for assessing PSQ. Data source: QUASR-R coding. Descriptive, descriptive assessment only; Inclusion, inclusion criteria; Pred/Mod, use of quality as a predictor or moderator; PubBias, publication bias; Sen/Sub, sensitivity/subgroup analysis. *N* = 117.

Figure 2 presents the proportion of articles in our sample that were associated with each of the purposes for assessing PSQ we coded. Among the 117 articles that reported PSQ assessments, 43 (36.8%) did so solely for the purposes of providing descriptive information in their meta-analyses. That is, these articles only described the outcomes of PSQ assessments, but did not discuss the implications of the assessment for how the results of the meta-analysis could be interpreted. Next, 26 (22.2%) articles in the subset reported PSQ as either a predictor or moderator in their meta-analysis, while 12 (10.3%) reported it as an inclusion criterion. Interestingly, 16 (13.7%) articles simply reported that PSQ was assessed, but did not describe the results of their assessments any further. It was therefore unclear why PSQ was assessed for these meta-analyses. Finally, 9 (7.7%) articles in our sample coded PSQ for multiple purposes. For most of these articles, quality was used as an inclusion criterion in conjunction with its use in subgroup or sensitivity analyses.

#### Survey

**FIGURE 3:**
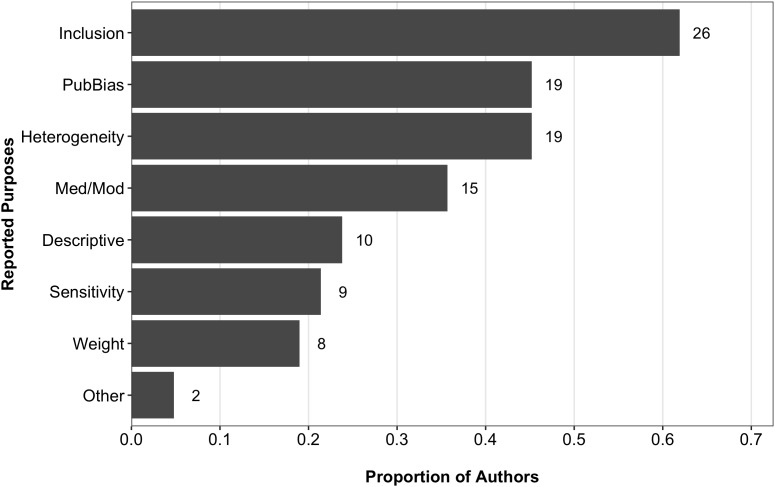
Proportions and frequencies of purposes authors cite for assessing PSQ. Data source: survey of first authors. Descriptive, descriptive assessment only; Inclusion, inclusion criteria; Pred/Mod, use of quality as a predictor or moderator; PubBias, publication bias; Sen/Sub, sensitivity/subgroup analysis. *N* = 42. Because authors were able to indicate more than one purpose, proportions do not sum to 1.

The subset of authors who indicated they assessed PSQ (*n* = 43) were asked to further indicate their purposes for assessing PSQ. A summary of their responses is shown in Figure 3. Compared to the coding findings, a larger proportion of authors indicated PSQ was assessed for the purpose of establishing inclusion criteria (26, 60.5%). Of these, only six were coded as reporting a PSQ assessment, leaving 20 articles that, at least in part, defined their inclusion criteria using PSQ without explicitly reporting it as such. Other notable differences between the coding and survey findings were observed when the purpose of assessing PSQ was to explore publication bias or to provide only descriptive information related to PSQ. With respect to the former, 44.2% (19 of 43) of authors indicated they used their PSQ assessments in their explorations of publication bias (e.g., as a predictor of bias), compared to the 1.7% (2 of 117) we coded as reporting that purpose. Of those 19, 12 did not report assessing PSQ in their published articles. Conversely, 23.3% (10 of 43) of authors responded to the survey as assessing PSQ for the purpose of providing descriptive information only, compared to the 36.8% (43 of 117) that were coded with the QUASR-R as such, with only three of those 10 coded as reporting their PSQ assessments. Although, 15 authors indicated using PSQ as a moderator or predictor, only two of those 15 (13.3%) reported this in the corresponding published meta-analysis. Finally, 19 (44.2%) of authors indicated using PSQ in their analyses of heterogeneity.^6^

### Criteria Used to Judge Primary Study Quality

**FIGURE 4:**
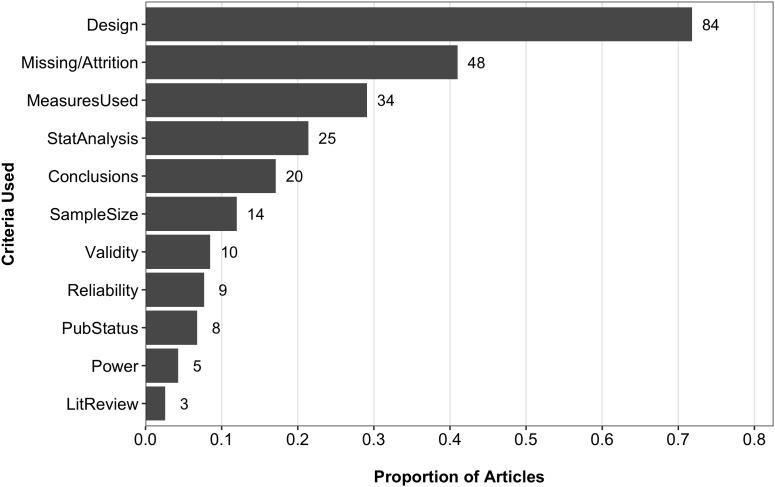
Proportions and frequencies of reported criteria used to judge PSQ. Data source: QUASR-R coding. Conclusions, adequacy of conclusions drawn in the individual primary studies; Design, study design of the individual primary studies; LitReview, literature review; MeasuresUsed, measures used in the individual primary studies; Missing/Attrition, missing data or attrition rates in the individual primary studies; PubStatus, publication status; StatAnalysis, statistical analyses used in the individual primary studies. *N* = 117. Because articles were coded for multiple criteria, proportions do not sum to 1.

The QUASR-R coded the following criteria for judging PSQ: the adequacy of conclusions made in the primary study, study design, information found in literature reviews, the measures used (e.g., instruments, scales, apparatus, etc.), missing data and attrition rates, power considerations, the publication status of the primary studies, statistical analyses used, sample size considerations, reliability estimates, and validity concerns. Figure 4 shows the proportion of articles coded as using the various criteria to judge PSQ from the 117 articles that reported PSQ assessments. Study design (84; 71.8%) and missing data/attrition rates (48; 41%) were the most frequently coded criteria. Other notable criteria included the measures used (34; 29.1%), statistical analyses used (25; 21.2%), the adequacy of conclusions drawn (20; 17.1%), and sample size considerations (14; 12%). The survey did not contain items related to specific criteria used to judge PSQ.

### Meta-Analytic Resources

In addition to the quality-specific survey items, two items related to the resources authors consulted when conducting and reporting their meta-analyses were examined.Authors were first asked to indicate which resources they consulted. The following response options were provided: (a) MARS; (b) Cochrane Handbook; (c) PRISMA; (d) QUOROM; (e) textbooks; (f) articles; and (g) other. Responding authors (*n* = 66) most frequently indicated textbooks (48; 72.7%), articles (50; 75.6%), and oftentimes both in conjunction (42; 63.6%). The remaining options were indicated at much lower rates, with the CH indicated by 33.3% of authors, the PRISMA by 19.7%, MARS by 10.6%, QUOROM by 7.6%, and other reference sources by 16.7%. The latter category included references to course notes and materials, reliance on the advice of experts or colleagues, and one mention of the Campbell Collaboration. The distribution for the utilization of resources is mostly similar between those who assessed PSQ and those that did not (see Table 2), the exceptions being that those who indicated they assessed PSQ utilized the CH and QUOROM at higher rates and textbooks and articles at lower rates. This survey item was followed by an item that asked authors whether any of the resources they indicated influenced what they reported in their published meta-analyses, of which 46 of 63 (73%) authors indicated that they had.

**Table 2:** Proportions of resources used by PSQ assessment.

	Study quality assessed		
Resource	Yes	No	Difference
MARS	0.103	0.111	–0.008
Cochrane	0.436	0.185	0.251
PRISMA	0.231	0.148	0.083
QUOROM	0.128	0.000	0.128
Textbooks	0.667	0.815	–0.148
Articles	0.718	0.815	–0.097
Other	0.154	0.185	–0.031


*Data source: survey of first authors. Example interpretation: 10.3% of authors that assessed PSQ indicated referencing the MARS when conducting and reporting their meta-analyses. MARS, Meta-Analysis Reporting Standards; Cochrane, Cochrane Handbook for Systematic Reviews and Interventions; PRISMA, Preferred Reporting Items for Systematic Reviews and Meta-Analyses; QUOROM, Quality of Reporting of Meta-analyses. *N* = 66. Because authors were able to indicate more than one resource, proportions do not sum to 1.*

### *Post hoc* Coding Findings

Following the planned analyses and subsequent findings for the QUASR-R items related to PSQ, additional QUASR-R items were examined *post hoc* in an attempt to gain insight about the possible factors that may have contributed to the underreporting of PSQ. First, given study design was by far the most utilized criteria for judging PSQ, the relationship between reporting PSQ and study design characteristics was examined. To this end, a crosstabulation of reporting rates of PSQ assessment with those of study design characteristics (see Table 3) showed that a majority of the meta-analyses that reported study design characteristics did *not* contain reports of PSQ assessments (157 of 255; 61.6%), leaving 98 articles (39.4%) that reported study design characteristics and PSQ as separate categories. We speculate that the conceptual overlap between these two categories may lead researchers to presume their reports of study design characteristics serve as *de facto* reports of PSQ, though we reserve further elaboration of this interpretation for the discussion.

**Table 3:** Design characteristics reported by PSQ reported.

	Study quality reported		
Design characteristics reported	Yes	No	Total
Yes	98	157	255
No	19	108	127
Total	117	265	382


*Data source: QUASR-R coding.*

**FIGURE 5:**
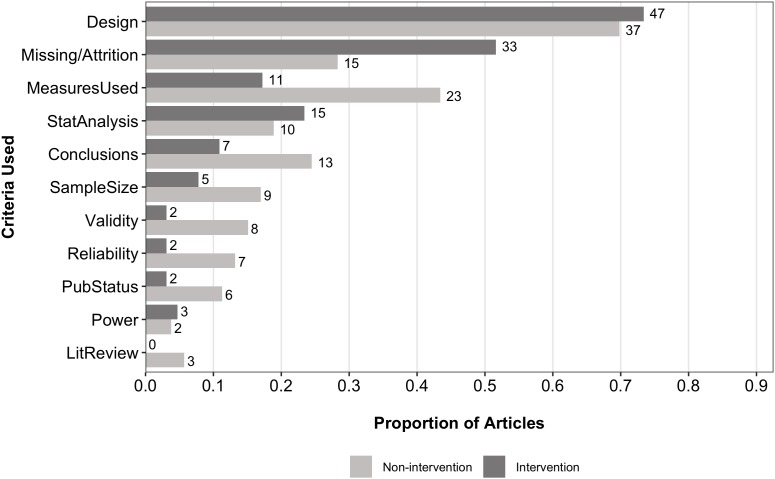
Comparison of criteria used to judge PSQ between intervention and non-intervention meta-analyses. Data source: QUASR-R coding. Conclusions, adequacy of conclusions drawn in the individual primary studies; Design, study design of the individual primary studies; LitReview, literature review; MeasuresUsed, measures used in the individual primary studies; Missing/Attrition, missing data or attrition rates in the individual primary studies; PubStatus, publication status; StatAnalysis, statistical analyses used in the individual primary studies. Sample sizes for intervention and non-intervention groups were 64 and 53, respectively. Because articles were coded for multiple criteria, proportions do not sum to 1.

Further, we hypothesized intervention-based meta-analyses were more likely to employ RCT designs and therefore assessments of PSQ would be more feasible for them due to their capability of using assessments tools designed to evaluate RCTs. Using the QUASR-R item that coded for research aim, we were able to categorize the coding sample into intervention (*n* = 116) and non-intervention meta-analyses (*n* = 266). Of the 116 intervention meta-analyses, 64 (55.2%) assessed PSQ in some form, whereas only 53 of the 266 (20.1%) non-intervention meta-analyses assessed PSQ. Comparisons between intervention and non-intervention meta-analyses on the other QUASR-R items revealed further differences. For example, comparisons between intervention and non-intervention meta-analyses showed that intervention studies tended to use missing data/attrition (51.6 vs. 28.3%) at a higher rate, study design (73.4 vs. 69.8%) and statistical analyses used (23.4 vs. 18.9%) at relatively similar rates, and all other criteria at lower rates than non-intervention studies (refer to Figure 5). We also found that 18 unique PSQ assessment tools were utilized among the 39 intervention meta-analyses that reported their assessment tool(s). For those 39, seven (17.9%) reported using the Jadad scale and 14 (35.9%) reported using the CH’s Risk of Bias Tool, with the remaining 16 assessment tools reported being utilized at frequencies of one or two intervention meta-analyses each. A similar pattern was observed for the 30 non-intervention meta-analyses, for which 20 unique assessment tools were employed. For those 30 meta-analyses, the NOS (6; 20%) and QUADAS (5; 16.7%) were the most prominently used assessment tools, with the remaining 18 tools utilized at frequencies of one or two non-intervention analyses. With respect to the purposes for assessing PSQ, the various purposes were reported at similar rates across both intervention and non-intervention meta-analyses. Regarding study design characteristics, when crosstabulations of reporting study design characteristics and PSQ were compared between the intervention and non-intervention meta-analyses, it was found that 40.7% of the 91 intervention meta-analyses that reported study design characteristics did not also report PSQ. An even higher rate was observed for the non-intervention meta-analyses (73.2% of 164; see Table 4).

**Table 4:** Study design characteristics by PSQ in intervention and non-intervention meta-analyses.

	Study quality reported					
	*Intervention*			*Non-intervention*		
	
Design characteristics reported	Yes	No	Total	Yes	No	Total
Yes	54	37	91	44	120	164
No	10	15	25	9	93	102
Total	64	52	116	53	213	266


*Data source: QUASR-R coding.*

Our final *post hoc* analysis involved examining more closely the 26 meta-analyses that reported assessing PSQ for the purpose of using it as a predictor or moderator. We found that PSQ was used as a moderator or predictor in 80 individual analyses contained within the 26 meta-analyses. Most often, PSQ was used as a categorical moderator or predictor (e.g., low-, medium-, high-quality) with 49 (61.25%) analyses conducted as such. PSQ was used as a continuous predictor or moderator (e.g., quality scale score) in the remaining 31 (38.75%) analyses. Interestingly, PSQ was not a significant moderator or predictor in the majority of the analyses (57, 71.25%). For PSQ moderators or predictors found to be significant, 12 showed a positive effect (i.e., higher quality corresponds to larger effect sizes), seven showed a negative effect (i.e., lower quality corresponds with larger effect sizes), and three showed a curvilinear effect when entered into a quadradic model. Twenty-five of the analyses were conducted by intervention meta-analyses and 55 conducted by non-intervention meta-analyses. Of the 24 analyses for intervention studies, most (17, 70.8%) were non-significant, with four significant negative effects and three significant positive effects.^7^ For the 55 analyses for non-intervention studies, a similar rate of non-significant moderators or predictors was observed (40, 72.7%), with nine significant positive effects, three significant negative effects, and three significant curvilinear effects. Finally, analyses for intervention studies used continuous PSQ moderators or predictors at a higher rate compared to analyses for non-intervention studies (60 to 29.1%, respectively).

## Discussion

The broad aim of the present study was to better understand the role of primary study quality in published psychological meta-analyses. Specifically, we aimed to describe reporting practices related to PSQ in psychological meta-analyses, as well as to further explore aspects of PSQ that might contribute to PSQ reporting and assessment by psychological researchers. In service of these aims, a two-phase approach was employed. The first phase consisted of an extensive coding project, for which a sample of 382 published meta-analyses was coded for quality-specific items, as well as for items related to other facets of conducting a meta-analysis. The following key questions were directly addressed: (a) Is PSQ generally reported? (b) If so, for what reported purposes is PSQ assessed? (c) By what criteria do psychological researchers judge PSQ? In addition to these questions, we explored how our quality-specific coding results related to other aspects of reporting a meta-analysis such as the reporting of study design characteristics, as well as how intervention and non-intervention meta-analyses compared across all findings. In the second phase, a survey was distributed to the first authors of the meta-analyses from the coding sample.Authors were able to directly indicate whether they assessed PSQ, what their purposes for assessing PSQ were, and the resources they consulted while conducting and reporting their meta-analysis. Taken together, the coding and survey data allowed us to examine the extent of overlap between researchers’ methodological practices and motivations and actual reporting.

Ultimately, the reporting rate of PSQ assessments was quite poor, with only 30.6% of our sample reporting that they had assessed PSQ in some way. Moreover, for those articles in which authors reported conducting some PSQ assessment, many did not report the procedures by which the assessment was conducted. Only 54.7% of the 117 articles that reported PSQ assessments explicitly described their coding procedures and only 6% of the 265 articles that did not include reports of PSQ provided some rationale as to why PSQ was not assessed. A comparison of intervention and non-intervention meta-analyses showed that reporting rates of PSQ were much higher among intervention meta-analyses at 55.2% (of 116) than non-intervention meta-analyses at 20.1% (of 266).

Initially we found these rates of reporting PSQ to be puzzling in light of the many published standards pertaining to reporting PSQ, including the American Psychological Association’s own MARS, as well as the PRISMA, CH, and others. Although it is difficult to come to any definitive conclusions as to why PSQ was so underreported in our sample, we suspect the answer to this question is multifaceted. One possible explanation is that assessments of PSQ are simply not conducted in psychological meta-analyses, that is, reporting a PSQ assessment is impossible if no such assessment was ever conducted in the first place. The survey of first authors attempted to explore this possibility by asking authors directly if they had conducted PSQ assessments for their meta-analyses. A majority of the sample of authors (43 of 71; 60.6%) responded that they had conducted some assessment of PSQ. However, when examining how many of those 43 authors’ published articles reported their PSQ assessments, it was found that only a minority (16; 37.2%) did so. This finding in particular lends some evidence to the hypothesis that PSQ is being assessed more often than the poor rate of reporting might otherwise imply. That being said, there remains a sizeable proportion of the sample of authors (39.4%) that did not assess PSQ in the first place.

That the sample of authors so often did not report PSQ assessments raises the question of whether researchers are aware that PSQ *should* be reported. Presumably, access to reporting standards such as the MARS, or other well-established resources would serve to inform authors of not only the importance of reporting PSQ assessments, but also the criteria used to judge PSQ and procedures employed for assessing it. Participant authors were asked about the resources they consulted while conducting and reporting their meta-analyses. The rates of utilization for the various reporting standards was found to be lower than one might expect. Particularly surprising was that, excluding the outdated QUOROM, the MARS was consulted least of all at a rate of 10.6% among the 66 responding authors. It was, however, not surprising that textbooks (72.7%), articles (75.6%), or both (63.6%) were the most frequently consulted resources, given they contain far more methodological content than do reporting standards. Sixty-three of those 66 authors responded to a follow-up item that asked whether the resources they consulted guided their reporting decisions, of which 43 (73%) indicated that they had. Seventy-nine percent of authors that used textbooks as a resource indicated that these influenced their reporting decisions. A similar rate of 78% was found for authors who indicated using articles as a resource. From these results it is clear that authors tend to rely much more heavily on textbooks and published articles to guide their reporting practices than they do on reporting standards.

It is possible that the apparent reliance on resources that are not explicitly designed to be reporting standards is in part attributable to the diversity of psychological research and consequently the diversity of reporting practices considered acceptable across research domains and publication outlets. It follows that researchers within a particular subdiscipline would look to past publications as indicators of what they should or should not report, both within their subdisciplines and within particular journals. Should that be the case, lapses in adherence to reporting standards could in part be due to the feedback loop inherent in such a strategy, wherein adherence or non-adherence in past publications encourages, by example, adherence or non-adherence in future publications. In this scenario, one might question the perceived *value* of PSQ to practicing researchers and publication outlets. By extension, one might also question whether the perceived value of PSQ contributes to the feedback loop that would be present if this scenario were true. In that vein, an interesting direction for future investigations would be to assess the degree to which reported assessments of PSQ moderate researchers’ trust in the precision and validity of meta-analytic findings. It follows that if assessments of PSQ have no bearing on how consumers of research interpret meta-analytic findings, then meta-analytic researchers may not consider assessing and reporting PSQ important and that reviewers for many publication outlets may not place a high value PSQ reporting. Whatever the reason, the above findings strongly suggest that reporting standards have not penetrated the field of psychology in a meaningful way, especially as they concern PSQ.

A final potential reason for underreporting considered here concerns the *concept* of PSQ and its relation to other distinguishable facets of conducting and reporting a meta-analysis. Namely, the specification of inclusion criteria and documentation of study design characteristics both share significant conceptual overlap with PSQ. Common among the three is a strong emphasis on study design. As previously discussed, many prominent methodologists conceptualize PSQ as pertaining primarily to the design of a primary study and the subsequent implementation of the study relative to its design. Regarding inclusion criteria, study design is often taken to be a foremost consideration. The CH, for example, notes the advantages of restricting the primary studies included in a meta-analysis to RCTs (see section 5.5 of Higgins and Green, 2011). The MARS also includes the documentation of study design features as a reporting item for both inclusion criteria and PSQ.

A number of our findings speak to the relationships between these three facets of conducting a meta-analysis. In the coding phase, frequencies were obtained for numerous criteria researchers reported using in their assessments of PSQ. By far, study design was the most frequently reported criterion at a rate of 71.8% among the 117 articles that reported PSQ. A crosstabulation of the reporting rate for PSQ against the rate for reporting study design characteristics showed that 157 of 255 (61.6%) articles reported study design characteristics but did *not* also report a PSQ assessment, compared to the 98 articles that reported study design characteristics and PSQ separately. With the exception of missing data/attrition being used at a higher rate and study design and statistical analyses used at similar rates, intervention meta-analyses used the remaining criteria at lower rates than non-intervention meta-analyses (see Figure 5). Further, non-intervention meta-analyses most often reported study design characteristics without also reporting PSQ (73.2% of 164), while intervention meta-analyses did so at a rate of 40.7% (of 91).

The purposes researchers reported for assessing PSQ were also coded. Assessing PSQ for purpose of specifying inclusion criteria was not often reported (12 of 117; 10.3%). Conversely, when we surveyed authors, a much higher proportion (26 of 43; 60.5%) indicated that their PSQ assessments were, at least in part, conducted for the purpose of defining their inclusion criteria. Most of these respondents, however, did not report PSQ in their published articles (20 of 26) and only one of the remaining six authors reported the purpose of their PSQ assessment as relating to inclusion criteria. Thus, as was the case for some of our other findings, a sizeable gap appears to exist between researchers’ motivations and their actual reporting practices. We suspect that because of the shared emphasis among PSQ, study design characteristics, and inclusion criteria on features related to study design, the three facets are sometimes conflated in reporting practice. Especially in conjunction with the under-utilization of reporting standards, we believe it is likely that researchers often fail to distinguish the three facets as separate reporting categories as they are presented in the MARS, PRIMSA, and CH. As such, it is possible that notions PSQ are implicitly “smuggled” into published meta-analyses via the reporting of inclusion criteria and study design characteristics. It may be the case that researchers believe their reports of inclusion criteria and/or study design characteristics are sufficient conduits for their PSQ assessments given the conceptual redundancy among these components of a meta-analytic synthesis, or perhaps, that any consideration of study design serves as PSQ assessment in and of itself. Paradoxically, the most utilized standards resource used by our sample of authors, the CH, goes to great lengths to separate the three facets. For example, the section on risk of bias explicitly states that any assessment of risk of bias is only to be conducted on those studies that are *already* included in a meta-analysis (Higgins and Green, 2011). Moreover, the CH makes clear that reports of study design characteristics should be supplemented with risk of bias outcomes as similar, but separate reporting items.

The concept of PSQ is also reflected in the priorities of published assessment tools. An assessment tool consisting primarily of items related to study design implies that study design is a primary component of the PSQ concept the tool intends to assess. Consequently, the plethora of published PSQ assessment tools may distort the PSQ concept. In our sample alone, only 69 meta-analyses reported using a particular assessment tool, yet among those 69 meta-analyses we found that 34 unique tools were used with most of the 34 tools being used within fewer than three meta-analyses. It is possible that the abundance of PSQ assessment tools discourages researchers from assessing or reporting PSQ. Researchers that intend to assess PSQ are required to select an assessment tool from the dozens of tools available to them and without an *a priori* conviction to a conceptualization of PSQ or to a particular tool, selecting a tool may be a paralyzing endeavor. Pragmatically speaking, the abundance of assessment tools may introduce an additional source of risk for researchers hoping to publish their work. With so many assessment tools available, there exists a chance that a researcher’s choice of tool may be considered a weakness of their study in the eyes of reviewers or editors. The selection of an assessment tool may therefore be perceived as a gamble to appease reviewers or editors to some researchers, which disincentivizes the assessment or detailed reporting of PSQ. Interestingly, a pattern of assessment tool preference was observed when comparing intervention and non-intervention meta-analyses. Intervention meta-analyses favored the Jadad scale and CH’s Risk of Bias tool, while non-intervention meta-analyses favored the NOS and QUADAS. Still, both groups reported using a wide array of scales, as previously mentioned.

The results of the coding phase of the present study suggests that the reporting practices related to PSQ are, and have been, suboptimal in the time since reporting standards were codified specifically for psychological meta-analyses by the MARS in 2008. Less clear, however, is an understanding of the mechanisms that drive and facilitate the practice of publishing meta-analyses without including information related to PSQ assessments. We contend that the present study has made some headway toward gaining such an understanding. We also acknowledge that the interpretations discussed in this section have been largely speculative. Unfortunately, such speculation is difficult to avoid when investigating reporting practices. However, the analogy between this circumstance and implications of our findings is not lost on us, that is, suboptimal reporting practices are detrimental to the interpretability of research findings. Just as the present study can only speculate about the unreported aspects of PSQ assessment (e.g., the purposes of assessments, the criteria for judging PSQ, the assessment tools used, etc.), so too can consumers of meta-analytic research only speculate about the validity of a meta-analytic finding in the absence of the full context in which the finding was produced. The significance of this implication cannot be overstated. The function of meta-analysis to synthesize collections of primary study outcomes into single-value estimates of summary effect makes it an unquestionably powerful tool in contemporary scientific practice. Meta-analytic methods are championed as offering higher degrees of precision, objectivity, and replicability than those obtainable by any primary study in isolation (Borman and Grigg, 2009). Moreover, as Cooper (2017) describes, “research syntheses focus on empirical research findings and have the goal of integrating past research by drawing *overall conclusions* (*generalizations*) from many separate investigations that address identical or related hypotheses” (p. 37, *emphasis added*). As such, the impacts of meta-analytic findings are far-reaching and carry with them an elevated presumption of scientific credibility. It is therefore crucial that reporting practices offer as much transparency regarding how a meta-analytic finding is obtained, so as to facilitate its clear interpretation, reproducibility, and ultimately to preserve the fidelity of meta-analysis as a powerful scientific tool.

### Limitations

A number of limitations beyond those already mentioned affected the present study. First, several relevant items from the QUASR-R were limited by their generality. That is, the subject matter that these items coded for was broadly defined. The generality of such items was necessary in order to maintain sufficient interrater agreement among the coders. As a consequence, some of the coding findings consist of a wide range of reporting practices. A second limitation concerns the sample sizes for the survey of first authors. While sample sizes exceeded 60 authors for most survey items, relative to the population of 382 first authors, the proportion of authors that did participate was rather low, ranging between 16.5 and 18.6% depending on the item. Some items were only shown to authors if they recorded a particular response on the previous item. Thus, sample sizes were further reduced for those follow-up items. For instance, only 43 of 71 authors responded that they had assessed PSQ and thus, the sample sizes for all follow-up items related to PSQ were limited to 43. Thus, we maintain a very conservative stance regarding the generalizability of the survey results. We view these as primarily providing some indicators of direction for future research. Finally, the scope of the present study encompasses many areas of psychological research, which may vary in their research practices. As such, the results reported in the present study should be interpreted in light of the variability in research aims and methodologies employed across the meta-analyses in our sample.

## Data Availability

The datasets analyzed for this study can be found in the Open Science Framework repository at https://osf.io/gqrs7/.

## Ethics Statement

This study was carried out in accordance with the recommendations of the Tri-Council Policy Statement 2 assessed by the Simon Fraser University Research Ethic Board with written informed consent from all subjects. All subjects gave written informed consent in accordance with the Declaration of Helsinki. The protocol was approved by the Simon Fraser University Research Ethic Board.

## Author Contributions

KS conceptualized the project and assisted in all phases of the project. DT led the first phase of the project, which involved co-authoring with KS the coding protocol used in the study, as well as supervising the empirical coding of the sample of published meta-analyses. RH led the second phase of the project, the responsibilities of which included co-authoring with KS and administering the survey to first authors. Data analysis was conducted by RH and DT. RH created the visualizations. RH, KS, and DT wrote the manuscript.

## Conflict of Interest Statement

The authors declare that the research was conducted in the absence of any commercial or financial relationships that could be construed as a potential conflict of interest.

## Footnotes

1 Panic et al. (2013) measured reporting quality as adherence to the PRISMA. Methodological quality was assessed using the Assessing the Quality of Systematic Reviews scale (AMSTAR; Shea et al., 2007).

2 Note that “primary study quality” refers explicitly to the quality of the individual studies included in a meta-analysis. This is not to be confused with the quality of a meta-analysis itself.

3 We understand the terms “exclusion criteria” and “inclusion criteria” to refer to the same process of selecting a sample of primary studies to from the collection of primary studies found in an initial search. The terms are treated as synonymous in this article.

4 Note that, with the exception of some clinical domains, the risk of bias conceptualization was not a well-established methodological concept within psychology during timeframe in which our sampled articles were published.

5 We do not mean to imply that the remaining 252 (94%) articles that did not report a PSQ assessment also did not conduct an assessment.

6 In the survey of first authors an additional “exploration of heterogeneity” response option was included for the question pertaining to the authors’ purposes for assessing PSQ, which did not reflect any item coded by the QUASR-R. A reviewer of this work has questioned how this response option differs from the response option of using PSQ as a predictor or moderator. At present, we do not recall what our rationale for including the extra heterogeneity option was at the time of drafting the survey. However, it is noteworthy that when the 19 articles whose authors endorsed the heterogeneity option were compared to the QUASR-R codes for reporting PSQ as a moderator or predictor, only two of the 19 articles reported a moderator analysis. The same two authors endorsed both the heterogeneity and moderator response options. We therefore believe that the two responses options were not confused by authors responding to our survey.

7 One of the 25 reported analyses for intervention studies did not report their statistical results and was therefore excluded from this tabulation.
